# Three donor site dressings in pediatric split-thickness skin grafts: study protocol for a randomised controlled trial

**DOI:** 10.1186/s13063-015-0557-9

**Published:** 2015-02-08

**Authors:** Craig A McBride, Roy M Kimble, Kellie Stockton

**Affiliations:** Pegg Leditschke Paediatric Burns Centre, Department of Paediatric Surgery, Lady Cilento Children’s Hospital, 501 Stanley Street, South Brisbane, QLD 4101 Australia; Centre for Children’s Burns and Trauma Research, Queensland Children’s Medical Research Institute, University of Queensland, 300 Herston Road, Brisbane, QLD 4029 Australia; Department of Paediatrics and Child Health, University of Queensland, Level 7, Lady Cilento Children’s Hospital, 501 Stanley Street, South Brisbane, QLD 4101 Australia

**Keywords:** Pediatric, Burns, Split-thickness skin graft, Donor site, Dressing, Randomised controlled trial, Pain, Scar, Itch, Re-epithelialisation

## Abstract

**Background:**

For children requiring split-thickness skin grafting for burn injury, the optimum donor site dressing is an ongoing subject of debate. The most common dressings in use, both regionally and worldwide, are calcium alginates. We will compare an alginate with two other dressings, all of which are in current use in the Pegg Leditschke Paediatric Burns Centre (PLPBC), to determine which dressing performs the best.

**Methods/Design:**

This is a randomised, prospective single center parallel three-arm trial comparing three donor site wound (DSW) dressings: Algisite™ M, a calcium alginate dressing; Cuticerin™, a smooth acetate gauze impregnated with water-repellent ointment (petrolatum, paraffin and Eucerite®) and Sorbact®, a gauze mesh coated with a dialkylcarbamoyl chloride (DACC) and amorphous hydrogel.

Primary outcomes are days to complete DSW healing, and pain. Previously validated measures will be used for all outcomes. Secondary outcomes are: itch; scar appearance at three, six and 12 months; ease of dressing application and removal and dressing costs and utility. Results will be analysed on an intention-to-treat basis. Donor site thickness will be measured with a small biopsy from the center of the graft, to document the depth of the DSW across the groups.

**Discussion:**

This study will provide comprehensive short- and long-term data on DSW dressings in pediatric split-thickness skin grafting. The best-performing dressing will become the preferred dressing for the PLPBC. We will provide rigorous data against which other dressings can be compared in future, recognising that alginates are the most common DSW dressing currently in use. Our study design replicates a real-world scenario in order to identify clinically significant differences between the three dressings.

**Trial registration:**

This trial was prospectively registered on 8 April 2014 with the Australia and New Zealand Clinical Trials Register (identifier: ACTRN12614000380695).

**Electronic supplementary material:**

The online version of this article (doi:10.1186/s13063-015-0557-9) contains supplementary material, which is available to authorized users.

## Background

Split-thickness skin grafting (STSG) is the most widely used method of achieving skin coverage in burns that are not predicted to heal rapidly with dressings. In harvesting skin to cover the defect, a second wound is created, the donor site wound (DSW). In children, this wound is predicted to re-epithelialise in seven to 14 days. The ideal DSW dressing does not adhere to the wound bed, facilitating removal. DSW dressings should be pain-free, reduce blood loss and be changed infrequently; ideally not until the wound has healed [1-4]. Among clinicians, a pain-free dressing is the most consistently desirable or essential quality of a wound dressing, in recognition of the fact that the DSW is often more painful than the recipient site [5]. The need for such a dressing is, if anything, more important in pediatric burn care as there are data suggesting that decreased pain leads to measurable and clinically significant improvements in wound healing [6-8]. Additionally, rapid healing permits the repeat harvesting of donor areas in larger burns and also decreases the risk of scarring [9].

The optimum dressing for the DSW is unclear, with surgeon and centre choice appearing to follow tradition stemming from eminence as often as it follows selection stemming from evidence. Alginates are the most commonly used DSW dressing [2,3]. There are four systematic reviews investigating DSW dressings [10-13], none of which have identified a single dressing with a clearly superior performance. Data are particularly lacking in pediatric burns, and new dressings are being developed continually. There is a clear need for high quality evidence in pediatric DSW dressings.

Due to the sparse data available, and in recognition of the variety of dressings in our unit, we aim to investigate DSW dressings currently in use in the PLPBC. This trial seeks to answer the following question: in pediatric STSGs, which of the three DSW dressings currently in use at our institution (Algisite™ M, Cuticerin™ and Sorbact®) is superior in terms of pain and itch, wound healing, ease of application and removal, scarring and costs? The best-performing dressing will become our preferred standard, against which we are likely to subsequently repeat the trial as newer dressings become available.

### The Pegg Leditschke Paediatric Burns Centre

The Pegg Leditschke Paediatric Burns Centre (PLPBC) is the major pediatric burns centre for Queensland and Northern New South Wales in Australia, serving a population of approximately 4.5 million. The PLPBC treats approximately 750 new burns annually. A total of 79 patients received split-thickness skin grafts in 2013. The majority of patients have small areas requiring grafting, and our preferred donor site is the thigh or buttock. On occasion grafts from the adjacent skin are used to combine donor and graft sites into a single dressing site. Facial burns are preferentially treated with scalp grafts whereas larger burns may require using the back as a donor site.

The PLPBC promotes ‘bench to bedside, and back to bench’ burns care. Clinical questions drive our research efforts, the result of which alter clinical care and in turn generate the next round of research questions. The centre has research and prevention arms, as well a clinical multidisciplinary team. The PLPBC has numerous publications in pediatric burns across many different aspects of this care; laboratory, clinical burns care, patient and family response to burn injury, prevention, rehabilitation, physio- and occupational therapy and procedural pain control and distraction modalities.

## Methods/Design

### Protocol and registration

This trial has approval from the Royal Children’s Hospital Human Research Ethics Committee (approval number: HREC/14/QRCH/36), and from the University of Queensland Medical Research Ethics Committee (approval number: 2014000447)*.* The methodology is specified in advance, documented in a protocol and has been registered prior to recruitment commencing (Australia and New Zealand Clinical Trials Register, identifier: ACTRN12614000380695) [14]. The methods are summarised here according to the revised Consolidated Standards for Reporting Trials (CONSORT) statement [15].

### Design and setting

**Figure 1:**
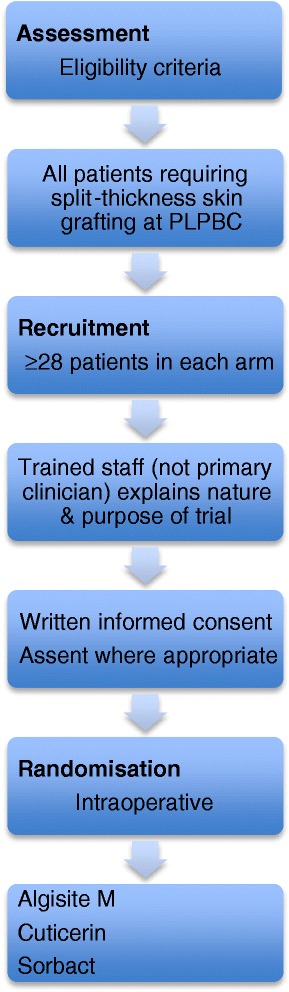
**Flow diagram of the trial.** DSW, donor site wound; PLPBC, Pegg Leditschke Paediatric Burns Centre.

We have designed a single centre prospective parallel randomised controlled trial with three treatment groups (Figure 1). There is no control arm in the generally accepted sense, but we recognise many will view alginates as a *de facto* control arm. Equipoise exists for this trial as there is no generally accepted DSW dressing among burns surgeons in general, and among the PLPBC surgeons in particular. We deem it unethical to have an inactive control arm as our purpose is to find which of three current dressings is superior.

The PLPBC will be moving to a new children’s hospital in the same city in the middle of this trial (expected date is late November 2014). All clinical staff will remain the same, and we have systems in place to manage this transition with no disruption to acute services. While this migration will be accompanied by a moratorium on elective surgery, this will not be the case for children enrolled in the trial and requiring acute surgery. Their operations and follow-up procedures and/or assessments will not be delayed or postponed due to the migration.

### Eligibility criteria for patients

From recruitment commencement, all patients of the PLPBC requiring STSG will be consecutively considered for inclusion, until recruitment is completed. Our unit treats only pediatric patients, aged from birth to 15 years. Regardless of the predicted size of the DSW, all children undergoing STSG will be considered for inclusion.

Patients will be assessed for eligibility by their primary surgeon or an investigator. If eligible, they will be approached by a trained staff member not involved in their primary care. Parent or caregiver informed consent will be obtained and documented. Where appropriate, child assent will be documented (Additional files 1 and 2).

Exclusion criteria include non-English speaking parents or caregivers, as we will not be able to gather data the following day by telephone. Also excluded are children with a known cognitive impairment, and children under the care or investigation of the Department of Communities, Child Safety and Disability Services. Only thigh or buttock DSWs will be included.

### Interventions

There is uniformity in the application of all three dressings within the PLPBC; all are dressings that are already in use. Three different DSW dressings will be used: Algisite™ M, an alginate dressing (Smith & Nephew, Hull, United Kingdom); Cuticerin™, a smooth acetate gauze impregnated with water repellent ointment (petrolatum, paraffin and Eucerite®) (Smith & Nephew, Hull, United Kingdom) and Sorbact®, a gauze mesh coated with a dialkylcarbamoyl chloride (DACC) and amorphous hydrogel (Abigo Medical AB, Gothenburg, Sweden). DACC is a synthetic fatty acid derivative. This dressing is designed to cause a hydrophobic interaction between bacteria and the dressing, lifting them from the wound bed in a moist environment [16].

We no longer routinely use open weave paraffin gauze or similar dressings directly on DSWs. The large mesh spaces in these dressings can lead to incorporation during re-epithelialisation which in turn can cause pain and bleeding on dressing removal. Further, leaving such a dressing on until spontaneous separation risks a permanent pattern on the DSW scar [17].

### Surgical procedure

The grafting procedure will be performed with a pneumatic dermatome (Zimmer Inc., Warsaw, Indiana, United States), according to local practice. The STSG will be harvested at between 0.005 and 0.008 of an inch (0.13 to 0.20 mm), according to surgeon preference and the age of the patient. It is anticipated that the majority will be taken with the dermatome set at 0.007 of an inch (0.18 mm), as this is the default local setting. The particular dermatome used will be recorded, as will the ‘swipe number’ of the dermatome blade in use at the time, the donor site and the surgeon harvesting the graft. A small lentiform biopsy will be taken from the center of each graft, blocked, cut and measured for graft thickness. Any remaining donor skin at the conclusion of the case will be similarly treated. This will allow more confidence regarding DSW depth when analysing results.

Following harvesting, local anesthetic (bupivacaine 0.25% with epinephrine 1:200,000 AstraZeneca Pty Ltd, North Ryde, NSW 2113, Australia) is applied directly onto the wound up to the maximum safe dose calculated from the weight of the child (2 to 2.5 mg/kg, depending on the child and any co-morbidities). Post-harvest topical application of local anesthetic was chosen rather than pre-harvest infiltration in order to further standardise the DSW, as infiltration was felt to be more variable. Topical local anesthetic gives good pain relief in DSWs [18]. In longer cases donor sites may be temporarily dressed with epinephrine and/or saline-soaked gauze (1 mg/1,000 mL NaCl), with or without pressure dressings, to facilitate hemostasis. The DSW is formally dressed at the end of the case, following dressing of the grafted site. One of the three dressings is applied directly to the wound; followed by an absorbent second layer of Allevyn™ and a Hypafix™ securement dressing (Smith & Nephew, Hull, United Kingdom). The majority of patients are sent home the same day.

### Wound treatment

Randomisation occurs immediately before the definitive DSW dressing is applied. Where space between donor sites permits (for example, between the left thigh and right thigh), a second randomisation is permitted. Each wound then constitutes its own study entity and will be analysed separately.

The DSW dressing is taken down in clinic on the seventh post-operative day. Our current standard dressing following this first dressing change is Xeroform® (Covidien, Mansfield Massachusetts, United States) 3% bismuth tribromophenate occlusive petrolatum gauze, which will be applied to all DSWs from this point. We expect the majority of wounds to be healed or nearly healed at seven days, from clinical observation. We have no experience using any of the three study dressings on a DSW beyond this first dressing change.

Clinics occur every weekday, and each patient is seen twice weekly after the first dressing change. This is predicated on ongoing treatment for the grafted site. By reviewing the donor dressing on these occasions we will avoid a requirement for additional clinic visits. Reviewing the dressings every three days after the first week replicates a real-world scenario and means that our results, if positive, will have clinical significance for this outcome.

### Co-interventions

Reinforcement of the primary dressing is permitted as per standard clinical practice. In such cases the reinforcing dressing, and the reason/s for applying it, will be recorded. On occasion children, parents or other health care providers outside the hospital remove the dressing within the first week. Should this occur, the study dressing will be reapplied and reasons for the early removal will be documented.

Interventions at dressing change, such as debridement, cleansing or coverage with cling-film prior to review are permitted in all arms, reflecting a real-life approach. In the case of a suspected or diagnosed wound infection the wound may be treated with antimicrobial agents (povidone iodine and/or nanocrystalline silver dressings). Common definitions for superficial surgical site infections (SSI) are predicated on an incisional, rather than excisional wound [19] and their appropriateness for DSW infection is questionable. We have taken a clinical approach based on the definition of a superficial SSI. Diagnosis of wound infection in this trial is by agreement of the treating clinician and a second burns surgeon. Criteria used for defining wound infection are qualitative, and include purulent discharge from the DSW surface; serous or haemoserous discharge alone does not constitute infection. Inflammation extending beyond the margins of the DSW at dressing change is another criterion. A surface swab is inaccurate and has a long lead time, but will nonetheless be performed for a colony count as it may provide some quantitative data in support of the diagnosis. A positive swab alone does not constitute an infection. We do not perform punch biopsies of our patients in clinic. As part of the dressing protocol these wounds will all be photographed.

Standard analgesia will be provided to all children. For larger total body surface area (TBSA) wounds it is our practice to augment this with gabapentin and/or antihistamines in an effort to decrease itch. These medications are surgeon dependent. All medications will be recorded.

### Study outcomes

#### Primary endpoints

There are two primary endpoints: pain, and time to re-epithelialisation. Pain will be assessed at several time points. Firstly, at four hours following completion of the operation. This will allow measurement in day-case patients prior to their discharge. By taking these scores face to face we hope to facilitate the telephone call the following day. It is also an opportunity to clarify directly with parents that we are scoring DSW pain, rather than graft site pain. Secondly, at one day later via a telephone call, or following ward review for inpatients. Thirdly, immediately before premedication prior to removal of the DSW dressing in clinic. Premedication will be with oxycodone 0.1 mg/kg orally (Mundipharma Pty Ltd, Sydney, NSW 2000, Australia), and oral paracetamol 150 mg/kg (sanofi-aventis Australia Pty Ltd, Macquarie Park, NSW 2113, Australia). Fourthly, at two minutes following removal of the DSW dressing. The graft dressing will not be taken down until after these scores are recorded. If a dressing is reapplied, the recordings at time points two, three and four above will be repeated for each subsequent change.

A variety of scales will be used for assessing pain, determined by who is performing the assessment (observer, parent or child), and by the age of the child. The face, legs, activity, cry and consolability (FLACC) scale will be used by nursing staff [20]. Where children are old enough, the Revised Faces Pain Scale (FPS-R) will be used for patient self-reporting of pain [21]. We will collect FPS-R data for children aged from three years, recognising that data are sparse for children below the age of five years [21]. Parents will use an 11-point (0 to 10) numeric rating scale (NRS). This, rather than a visual analogue scale, was chosen to facilitate the accuracy of recording during a telephone call.

Re-epithelialisation is defined as over 95% wound healing and no requirement for further wound dressing. Scabs or crusts are defined as unhealed areas. This endpoint is doubly assessed. In the clinic the treating doctor assesses percentage re-epithelialisation. It is further assessed from digital photography by an independent surgeon blinded to the DSW dressing. Photographs will be taken at each dressing change and at each subsequent clinic visit for care. In the clinic a visual percentage re-epithelialisation will be requested. The blinded observer will subsequently mark out the edges of the wound, and any unhealed areas. Surface area computer mapping will then be used to determine percentage re-epithelialisation. We will test concordance between clinician assessment and computer mapping regarding re-epithelialisation.

#### Secondary endpoints

Itch is a secondary endpoint. The Itch Man Scale will be used for assessing itch. This scale has been validated in children [22]. These assessments will occur at the same time as the pain scores above. Itch will also be assessed at the three, six and 12 month reviews. DSW appearance at three, six and 12 months is a secondary endpoint. This will be assessed in a number of ways: ultrasound measurement of scar thickness; digital three-dimensional photography and edge marking to determine computer-mapped areas of scarring. This will be compared with previous digital images; Patient and Observer Scar Assessment Scale (POSAS) [23]. We chose POSAS as this has superior performance in a systematic review over other scar assessment scales, and from a pragmatic viewpoint is the scale PLPBC staff are most familiar with [24]. It also seeks the opinions of the patient regarding their scar, something we consider important. In practice for many of our young children we use the parent opinion. Where possible we ask both parent and patient. There are some reliability data supporting the use of this scale in children above the age of six years [25]. Below this age caregiver opinion will be sought as a proxy for the child.

Ease of dressing application and removal is a secondary endpoint. Setup time will not be recorded; times for removal and reapplication of dressing will be recorded. A numeric rating scale (0 to four) will be used to ascertain opinions regarding ease of dressing removal at first change. Ease of dressing measurements in theatre are confounded by lack of blinding, and by the variable nature and size of the areas to be dressed. Ease of dressing scores in clinic will focus primarily on the ease with which the primary dressing is removed from the skin surface.

Cost is a secondary endpoint. This will be assessed using hospital pricing for the dressings used, and nursing time for dressing changes. Additional visits, where required, will also be included in the cost analysis. Routine visits will not be costed, since these are a standard part of treatment. Any additional treatments outside of standard care, such as treatment for infection, will be included in costing. A cost-utility analysis will not be performed, as the site and nature of the burn wound grafted will confound quality of life results. We will thus not be able to isolate the DSW from the grafted site in order to perform cost-utility on the DSW alone.

### Randomisation

Randomisation will occur in the operating theatre, immediately prior to application of the DSW dressing. We have used unrestricted randomisation, generated using an online program (http://stattrek.com/) [26]. Randomisation of participants will continue until each of the study groups has a minimum of 28 participants reaching the primary endpoints. Allocation concealment will be via the use of sequentially numbered, sealed opaque envelopes pre-prepared by a third party.

Patients will be recruited into the trial when a decision to graft is made following review of the burn injury. The patient will be randomised intra-operatively, further separating recruitment and consent from randomisation.

### Blinding

The nature of the study mitigates against full blinding, but some assessments can be blinded. Secondary dressings for all patients are the same, so clinic staff will not initially know which arm the patient is in. Clinic staff, patients and parents are blinded for assessments of pain and itch until the first dressing change. Following that point, blinding is not possible for the remainder of the pain and itch assessments as the dressing will have been revealed.

One of the assessors of re-epithelialisation will be blinded, as these assessments will take place using digital photography. Details of the dressing will not be made available to this assessor. Where there is discrepancy between these two observations, the blinded assessor will be taken as the definitive assessment in an effort to overcome performance bias. The assessments of scar appearance will also be blinded, as assessors will have no knowledge of the dressing used.

### Sample size

The sample size calculation was derived from the primary outcomes. Dressings are changed every three days after the first week, and we have used three days as a minimum clinically important difference (MCID) for re-epithelialisation. The standard deviation (SD) on DSW healing, from our database, is four days. We have chosen to power the study to 80%, with an α of 0.05, requiring 28 participants in each group. This sample size is also adequate to determine an MCID of 2 (SD 2.5) in our pain scores using the FLACC and the NRS.

We will use simple randomisation in order to maintain the same degree of DSW dressing unpredictability for each subject. Recruitment will continue until there are at least 28 participants in each arm with primary outcome data. A two-year recruitment period is anticipated, based upon the number of eligible patients requiring grafting in our unit in previous years.

### Data collection

A series of standardised case record forms have been designed using Filemaker Pro 13 (Filemaker Inc., Santa Clara, California, United States). These are loaded onto tablets for use in theatre and in the clinic. These tablets are a standard part of our data collection system already in place, and staff are trained in their use.

Data are collected on baseline demographic and patient condition characteristics, as well as the interventions used. Clinic and operative databases will be married to the study database. Data entry will be by clinic staff in the course of patient treatment. Discrepancies will be resolved by discussion and by re-checking the data. Where consensus is not possible that field will be left empty.

### Data monitoring

Data are monitored weekly for completeness, and reminders are sent to capture incomplete data. All researchers spend a considerable amount of time in the clinic and theatre, and will be closely associated with data collection. We currently capture data on all patients seen in clinic; some of which is submitted to the Australia and New Zealand Bi-national Burns Registry (ANZ Bi-NBR) [27]. The data we collect are more detailed than required for the ANZ Bi-NBR. Our current data completeness rate for our databases is 100%. The dressings we use are already a part of current practice in our unit; therefore we have not set up a data safety monitoring board for adverse effects. Our unit already has a robust monitoring system, with a weekly team meeting and a monthly morbidity meeting.

### Data analysis

All statistical analyses will be conducted using SPSS version 22 (IBM Corporation, Armonk, New York, United States). Demographic data will be analysed through descriptive statistics and between-groups analyses using analysis of variance (ANOVA) and/or non-parametric equivalents where appropriate. Generalised linear models, estimating variance appropriately for repeated measures where required, will be used to determine whether there are differences between groups in primary and secondary outcomes. Changes in the intervention effects will be examined with thickness of split skin graft, donor site surface area and ease of dressing removal considered *a priori* to be of potential interest. All data will be analysed as intention to treat (ITT) and per protocol, with ITT considered the primary method. All tests will be two-tailed and only those with a *P* value <0.05 will be considered statistically significant.

### Data storage

Data are backed up to a master file on a University of Queensland server. This file is password protected, and only available to the named researchers on the Human Research Ethics Committee (HREC) approvals. On completion of the project, identifying data will be removed from the record. The record will be kept in perpetuity in order that these data may form a historical cohort if the study is repeated with future dressings. De-identified data may be made available to other researchers in future to facilitate systematic reviews or meta-analyses. If such data are requested, they will only be made available following HREC review and approval.

## Discussion

For such a commonly created wound, it is surprising that there is no uniform dressing choice for DSWs [2,3]. There are a number of possible reasons for this: multiple dressings may have equivalent efficacy; more attention is focused on the grafted site, and the DSW is seen as a secondary priority and there are a lack of reliable and valid data regarding dressing choices.

While there are a number of trials and systematic reviews, heterogeneity between studies makes direct comparisons difficult [10-13]. Tentative recommendations support the use of dressings designed to create a moist healing environment. The majority of studies have been performed in adults. While it is tempting to extrapolate adult data to a pediatric setting this may not be entirely appropriate. Children’s skin is different to adults, and its thickness varies by site, age and gender [28], therefore trials in children may not have the same results as those in adults.

PLPBC DSW dressings are determined largely by surgeon preference and dressing availability on the day of surgery. For external readers this trial will provide data for two dressings against the active control of a calcium alginate. With rigorous trial design, we hope to be able to re-use this protocol in future should additional dressings become available, which may offer advantages over our chosen DSW dressing.

Our trial is intentionally pragmatic; we aim to replicate current clinical practice as closely as possible. We do not, for example, impose additional visits on participants. We are looking for outcomes that are clinically significant, and have chosen endpoints that are important to clinicians, and to patients and their families. Where possible we have used blinded assessments to second-read decisions in clinic. The scales we have chosen have been previously validated in children, and we have experience in all scales that will be used in this trial. This is the first trial of DSW wound healing that incorporates measurement of the harvested graft thickness into the study protocol. This will enable us to correlate healing with donor thickness.

Pain is a primary outcome of interest to parents and clinicians alike, as well as to the patient. Data are mixed on donor site pain, but the majority of studies do agree that DSW pain is often more severe than graft site pain. This is not a surprising finding given the nature of the two wounds. A donor site more painful than its corresponding recipient site has an associated eponym; a positive Moriarty sign is said to be a reassuring sign of impending graft take, rather than graft failure [5].

Time to healing is a relevant primary outcome. Faster re-epithelialisation allows us to re-harvest the donor site, as well as limiting the timespan of this iatrogenic injury. We try where possible to avoid meshed grafts, as the final appearance is cosmetically inferior.

There are a paucity of data on the long-term appearance of a DSW. Much scar studies have focused on the grafted site and DSW scars as well. The Queensland climate is warm, and children often have their thighs uncovered. In our climate the DSW may be more on view than the grafted site itself, as our most common burn injury is a scald to the anterior chest. Scar appearance is a secondary outcome. Scarring is related to the depth of injury, so to accommodate this variable we will be measuring the depth of the STSG at each donor site.

## Trial status

The first patient was randomised intra-operatively on 17 April 2014. As of 1 August 2014 we had recruited 36 patients. A total of 34 have been grafted and randomised to one of the three dressings, with a further two awaiting surgery.

## Additional files

Additional file 1:
**Child Assent form. Child assent form for older children involved in the trial.**
Additional file 2:
**PICF for Parent Guardian v3. Parent/guardian information and consent form.**

## Abbreviations

ANOVA: Analysis of variance
ANZ BiNBR: Australia and New Zealand Bi-National Burns Registry
CCBTR: Centre for Children’s Burns and Trauma Research
CONSORT: Consolidated standards of reporting trials
DACC: Dialkylcarbamoylchloride
DSW: Donor site wound
FPS-R: Revised Faces Pain Scale
FLACC: Face, Legs, Activity, Cry, Consolability pain scale
HREC: Human research ethics committee
ITT: Intention to treat
MCID: Minimum clinically important difference
NRS: Numeric rating scale
POSAS: Patient and Observer Scar Assessment Scale
RCT: Randomised controlled trial
SD: Standard deviation
PLPBC: Pegg Leditschke Paediatric Burns Centre
SSI: Surgical site infection
STSG: Split-thickness skin graft
TBSA: Total body surface area
