# Supplementary material for: Three donor site dressings in pediatric split-thickness skin grafts: study protocol for a randomised controlled trial
Source: Trials. 2015 Feb 8;16:43. doi: 10.1186/s13063-015-0557-9 (PMC4335760; doi:10.1186/s13063-015-0557-9)
Supplement: Additional file 1: — Child Assent form. Child assent form for older children involved in the trial. [file 13063_2015_557_MOESM1_ESM.docx]

Hello ……………………………………………………………………………

I work at this hospital and I want to know if we can help make your burn wound get better quickly.

- Is it OK for me to talk to you, and ask you to point to some faces to let me know how you are feeling when you are getting your bandages off and on?
- Is it OK if I take a photo of your donor site, so that I can see how quickly it gets better?
- Is it OK if you come back to see me at the hospital in a few months so that I can have a look at your skin where the donor site was, and take another photo?

If you change your mind and you don’t want to point to the faces any more or have the photos taken, then that would be ok too.


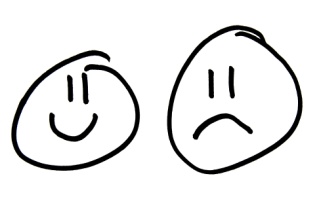


Can you circle (or colour in) for YES, and


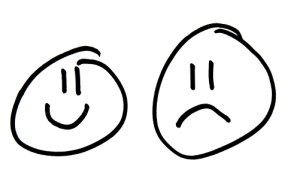


for NO

on the back of this page to tell us if it’s ok?

Thanks

My name is


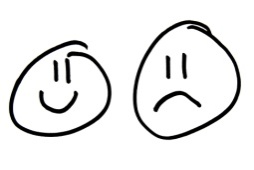

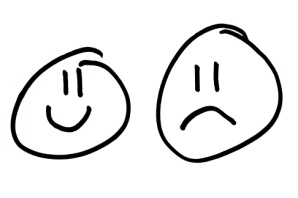


| I am happy to talk to you | 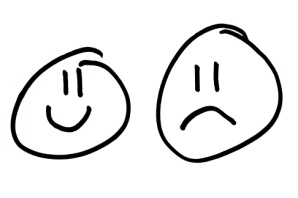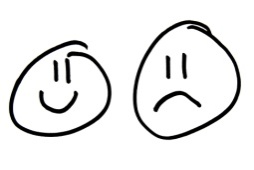 |  |
| --- | --- | --- |
| I’m happy to point to the funny faces when you ask me to | 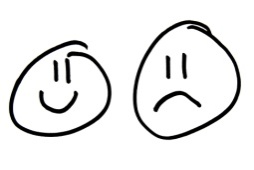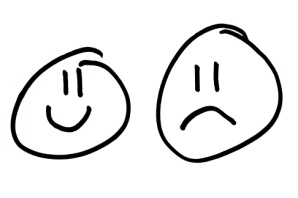 |  |
| I’m happy for you to take a photo of my burn | 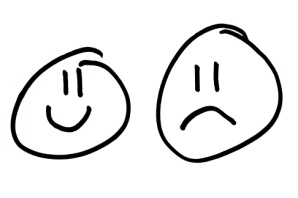 |  |
| I’m happy to come back and see you in a few months and have a photo taken of my skin | 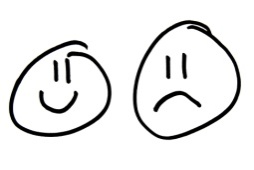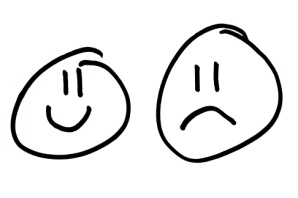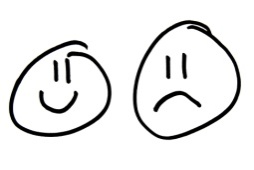 |  |
| I know it’s ok for me to change my mind |  |  |

My Signature
