# Supplementary material for: Three donor site dressings in pediatric split-thickness skin grafts: study protocol for a randomised controlled trial
Source: Trials. 2015 Feb 8;16:43. doi: 10.1186/s13063-015-0557-9 (PMC4335760; doi:10.1186/s13063-015-0557-9)
Supplement: Additional file 2: — PICF for Parent Guardian v3. Parent/guardian information and consent form. [file 13063_2015_557_MOESM2_ESM.doc]

**Participant Information Sheet/Consent Form - Parent/Guardian**

Royal Children’s Hospital, Brisbane

| **Title** | A prospective randomised controlled trial comparing donor site dressings in children requiring split skin grafting following burn injury |
| --- | --- |
| **Protocol Number** | Version 3 (dated March 19, 2014) |
| **Principal Investigator** | Dr Craig McBride  Centre for Children’s Burns & Trauma Research  Dept Paediatrics and Child Health, Royal Children’s Hospital  Level 3, Foundation Bldg, Herston 4029  (07) 3636 8111 |
| **Associate Investigator(s)** | Dr Kellie Stockton, Dr Roy Kimble |

Please make sure you have read and understood all 6 pages before signing the Consent Form to participate. The assent form is also to be filled out by the child if able.

**Part 1 What does the child’s participation involve?**

**1 Introduction**

**Participation in this research is voluntary. If you do not wish for your child to take part, they do not have to. They will receive the best possible care whether or not they take part.**

This is an invitation for the child in your care to take part in this research project: A prospective randomised controlled trial comparing donor site dressings in children requiring split skin grafting following burn injury. The research project is aiming to compare three different commercially available dressings used on donor site wounds in children.

This Participant Information Sheet/Consent Form tells you about the research project. It explains the tests and research involved. Knowing what is involved will help you decide if you want your child to take part in the research.

Please read this information carefully. Ask questions about anything that you don’t understand or want to know more about. Before deciding whether or not your child can take part, you might want to talk about it with a relative, friend or local doctor.

If you decide you want your child to take part in the research project, you will be asked to sign the consent section. By signing it you are telling us that you:

• Understand what you have read

• Consent to your child taking part in the research project

• Consent to your child having the tests and research that are described

• Consent to the use of your child’s personal and health information as described.

You will be given a copy of this Participant Information and Consent Form to keep.

**2 What is the purpose of this research?**

The goal of burn wound healing is to promote early healing as this has considerable influence on the long term quality and appearance of a scar. Children often require ongoing scar reconstruction operations throughout childhood. It is therefore important that the initial care of the burn wound is optimum to assist wound healing and minimise scarring. Sometimes skin grafting is required to help a burn heal. A thin layer of skin is taken from another part of the body, usually the thigh (this is called the donor site) and placed over the burn area. The donor site wound is like a graze, dressings are required over the donor site to help the wound heal.

Despite the large number of dressings available on the market, very few good studies have been carried out. The aim of this study is to compare three different commercially available donor site dressings in children. These are the three dressings we currently use in our Burns Unit.

**3 What does participation in this research involve?**

Your child will be participating in a randomised controlled research project. Sometimes we do not know which treatment is best for treating a condition. To find out, we need to compare different treatments. We put people into groups and give each group a different treatment. The results are compared to see if one is better. To try to make sure the groups are the same, each participant is put into a group by chance (random). This research project has been designed to make sure the researchers interpret the results in a fair and appropriate way; and avoids study doctors or participants jumping to conclusions.

Following signing the consent form your child will be randomly assigned to one of three groups. Each group will have a different dressing applied to donor sites. There are no new or experimental dressings used in this study – all three combinations are used in burns units in Australia. Information including age, ethnicity, cause of injury, percentage body surface area involved, and medication taken will be documented for your child. This information will remain confidential and accessible only to those directly involved in your child’s medical care or this research project. Dressings will be changed one week following surgery in the burns outpatient department.

At the dressing change a photograph of the donor site will be taken. Specific questionnaires/scales will be used to determine pain related to the dressing change. Ratings will be given by the nurse, yourselves, and by your child before and after wound dressing removal.

So that we can see how well your child’s skin heals we would like you to come back to the hospital 3 and 6 months after your injury. At this time we will be taking more photos and measuring the thickness of your child’s skin using an ultrasound machine. The ultrasound is not painful. We put some warm gel on the skin and run a small smooth transducer (wand) gently over the surface of the skin.

You will not be charged anything for taking part in this study, nor will you be paid for taking part.

**4 What does the participant have to do?**

Participation in this study will not alter the management of your child’s burn in any way other than the dressing applied to the wound. All 3 dressing combinations used in this study are routinely used in various burns units throughout Australia. In order for the researchers to assess the donor site we ask that children are followed up by the researchers 3 and 6 month following healing. Appointments will be made at a convenient time for you and your child, and at a time to correspond with visits to the Doctor and/or occupational therapist.

**5 Other relevant information about the research project**

A total of 93 children will be recruited to this study (31 in each dressing group). There is no inactive control/placebo group. What we mean by this is that there is no group that will receive no treatment – all of these treatments are dressings we currently use, we’re just trying to find out which is best. All children involved in this study will be recruited from Royal Children’s Hospital, Brisbane.

**6 Does your child have to take part in this research project?**

No. The decision to take part in this or not is entirely yours. If you do not wish for your child to take part, they do not have to. If you decide that they can take part and later change your mind, you are free to withdraw them from the project at any stage.

If you do decide that your child can take part, you will be given this Participant Information and Consent Form to sign and you will be given a copy to keep.

Whatever you decide, that’s ok with us. Either way we will still give your child the best care we can for their burn – they’re too important for us not to do that.

**7 What are the alternatives to participation?**

Your child does not have to take part in this research project to receive treatment at this hospital. If you choose not to participate in this study your child will receive exactly the same standard of care as all other patients who enter the burns centre.

**8 What are the possible benefits of taking part?**

There are about 800 children with new burns seen by us each year. The information you and your child give us about their dressings will help us to work out which is the best dressing – so that we can use that one as our preferred dressing in the future.

**9 What are the possible risks and disadvantages of taking part?**

There are none that we can identify because we are using each of these dressings at the moment, and your child would have had their wound dressed with one of them anyway.

**10 What if new information arises during this research project?**

Sometimes during the course of a research project, new information becomes available about the treatment that is being studied. If this happens, one of the researchers will tell you about it and discuss with you whether you want your child to continue in the research project. If you decide to withdraw the participant, the researcher will make arrangements for their regular health care to continue. If you decide your child is to continue in the research project, you will be asked to sign an updated consent form.

Also, on receiving new information, the study doctor might consider it to be in the child’s best interests to withdraw from the research project. If this happens, he/ she will explain the reasons and arrange for the participant’s regular health care to continue.

**11 Can your child have other treatments during this research project?**

Participating in this study will not affect any other treatments the child is having. It is important to tell the study doctor and the study staff about any treatments or medications your child may be taking, including over-the-counter medications, vitamins or herbal remedies, acupuncture or other treatments. You should also tell the study doctor about any changes to these during the child’s participation in the research project.

**12 What if your child is withdrawn from this research project?**

If you decide to withdraw from this research project, please notify a member of the research team. Your decision to withdraw from the study will not affect routine medical treatment or your relationship with staff of the Stuart Pegg Paediatric Burns Unit at the Royal Children’s Hospital.

**13 Could this research project be stopped unexpectedly?**

As the dressings used in this study are already in use clinically it is unlikely that this project will be stopped unexpectedly.

**14 What happens when the research project ends?**

When your child completes all aspects of this study (after the 6 month donor site assessment) there will be no further requirements in relation to this study from you or your child. A brief report outlining research findings will be emailed to you with your permission at the completion of the study in late 2015.

**Part 2 How is the research project being conducted?**

**15 What will happen to information about your child?**

By signing the consent form you consent to the study doctor and relevant research staff collecting and using personal information about your child for the research project. Any information obtained in connection with this research project that can identify your child will remain confidential. All information collected as part of this study will be stored safely in a locked filing cabinet in a locked office and password protected computer files. The figures we record from your child, eg demographics and questionnaire results, will be stored under a study number not an actual name. Any form that can identify your child, such as the consent and assent forms, will be stored separately to the other information in another locked office. This information from the study will be kept for 15 years in accordance with hospital policy. It will then be destroyed in a confidential shredder and using the latest data cleaning software. Only the investigators in this study can access this information. Your child’s information will only be used for the purpose of this research project and it will only be disclosed with your permission, except as required by law.

Information about your child may be obtained from their health records held at this and other health services, for the purpose of this research. By signing the consent form you agree to the research team accessing health records if they are relevant to participation in this research project.

We anticipate that the results of this research project will be published and/or presented in a variety of forums. In any publication and/or presentation, information will be provided in such a way that the participant cannot be identified. A study number only will be used and the information is presented in journals or conferences as group results, that is an average of figures obtained rather than individual results.

Information about participation in this research project may be recorded in your child’s health records.

**16 Who is organising and funding the research?**

This research project is being conducted by Dr Craig McBride, one of the surgeons in the Burns Unit. The study is supported by a research grant by Abigo. This company makes one of the dressings. However, the study was initiated and designed by the investigators, Abigo had no input into study design. They also have no access to the information we gather, and they will not have any part in the study, the analysis, or the writing of the report for publication.

**17 Who has reviewed the research project?**

All research in Australia involving humans is reviewed by an independent group of people called a Human Research Ethics Committee (HREC). The ethical aspects of this research project have been approved by the HREC’s of The Royal Children’s Hospital and the University of Queensland.

This project will be carried out according to the *National Statement on Ethical Conduct in Human Research (2007)*. This statement has been developed to protect the interests of people who agree to participate in human research studies.

**18 Further information and who to contact**

Clinical contact person

| Name | Kellie Stockton |
| --- | --- |
| Position | Clinical Research Manager  Centre for Children’s Burns & Trauma Research |
| Telephone | (07) 3636 1278 |
| Email | k.stockton@uq.edu.au |

Should you wish to discuss the study with someone not directly involved, in particular, any matters concerning policies, information about the conduct of the study or your rights as a participant, or you wish to make a confidential complaint, at any time, you may contact the Co-ordinator of the Ethics Committee on 3636 9167. If this phone is unattended, please leave a message and your call will be answered as soon as possible.

| Reviewing HREC name | Children’s Health Services Human Research Ethics Committee |
| --- | --- |
| HREC Co-ordinator | Amanda Smith |
| Telephone | (07) 3636 9167 |
| Email | AmandaJ.Smith@health.qld.gov.au |

**Reviewing HREC approving this research**

| Reviewing HREC name | University of Queensland Human Research Ethics Committee |
| --- | --- |
| Ethics Officer | Michael Tse |
| Telephone | (07) 3365 3924 |
| Email | humanethics@research.uq.edu.au |

Local HREC Office contact (Single Site -Research Governance Officer)

| Name | Peng Tjun Choy |
| --- | --- |
| Position | Research Governance Officer |
| Telephone | (07) 3636 4445 |
| Email | Peng_Tjun_Choy@health.qld.gov.au |

**Consent Form – Parent/Guardian**

| **Title** | A prospective randomised controlled trial comparing donor site dressings in children requiring split skin grafting following burn injury. |
| --- | --- |
| **Protocol Number** | Version 2 (dated February 26, 2014) |
| **Investigators** | Dr Craig McBride, Dr Kellie Stockton, Prof Roy Kimble |
| **Location** | Children’s Health Services Queensland |

**Declaration by Parent/Guardian**

I have read the Participant Information Sheet or someone has read it to me in a language that I understand.

I understand the purposes, procedures and risks of the research described in the project.

I have had an opportunity to ask questions and I am satisfied with the answers I have received.

I freely agree to the child participating in this research project as described and understand that I am free to withdraw them at any time during the project without affecting their future health care.

I understand that I will be given a signed copy of this document to keep.

|  |  | |  | | | |  |
| --- | --- | --- | --- | --- | --- | --- | --- |
|  | Name of Child (please print) |  | | | | |  |
|  |  |  | | | | |  |
|  |  | |  | |  |  |  |
|  | Name of Parent/Guardian (please print) | | |  | | |  |
|  |  | | |  | | |  |
|  | Signature of Parent/Guardian | |  | | Date |  |  |
|  | | | | | | | |

**Declaration by Investigator**

I have given a verbal explanation of the research project, its procedures and risks and I believe that the parent/guardian of the participant has understood that explanation. I have provided the parent/guardian with a copy of the participant information sheet.

|  | | | | | | |
| --- | --- | --- | --- | --- | --- | --- |
|  | Name of Investigator (please print) | |  | | |  |
|  | | | | | |  |
|  | Signature |  | | Date |  |  |
|  | | | | | | |

**Independent Witness**

I have witnessed the receipt of a Patient Information Sheet by the parent/guardian and exchanging of information between the investigator and the parent/guardian about the study.

|  | | | | | | |
| --- | --- | --- | --- | --- | --- | --- |
|  | Name of Witness (please print) | |  | | |  |
|  | | | | | |  |
|  | Signature |  | | Date |  |  |
|  | | | | | | |

Note: All parties signing the consent section must date their own signature.
